# Drug-Induced Liver Injury After Zoledronic Acid Infusion and Literature Review

**DOI:** 10.1210/jcemcr/luaf046

**Published:** 2025-03-20

**Authors:** Christopher Boldt, Pooja Prasad, Huifang Lu

**Affiliations:** Baylor College of Medicine, 1 Baylor Plz, Houston, TX 77030, USA; Baylor College of Medicine, 1 Baylor Plz, Houston, TX 77030, USA; UT MD Anderson Cancer Center, 1515 Holcombe Blvd, Houston, TX 77030, USA

**Keywords:** zoledronic acid, liver injury, osteoporosis

## Abstract

Zoledronic acid, a bisphosphonate, is commonly used to treat and prevent osteoporosis. Here we report a rare case of acute hepatotoxicity after zoledronic acid infusion in a 50-year-old female patient with no preexisting liver disease taking anastrozole and abemaciclib. Only a few hours after her infusion, the patient developed severe body aches, nausea, and abdominal bloating. Laboratory tests revealed an acute liver injury. Acute viral hepatitis workup was negative, and she was subsequently diagnosed with a hepatocellular drug-induced liver injury. Her transaminitis downtrended over the following day and normalized after 11 days. Given this rarely reported side effect, there is a paucity of evidence to guide treatment decisions. The goal of this case report is to increase awareness of this adverse effect, especially among patients taking other potentially hepatotoxic drugs.

## Introduction

While aromatase inhibitors have revolutionized breast cancer treatment, prolonged use is associated with side effects, such as bone loss and increased fracture risk [1]. Zoledronic acid (ZOL), a bisphosphonate (BP) given intravenously, has been approved for treating aromatase inhibitor-induced osteoporosis. The overall safety of BP treatment is well established. The most common adverse effects are flu-like symptoms after ZOL infusion, including fever, chills, myalgia, and arthralgia. BP-induced liver injury is rare, and specifically with ZOL, there have been only 5 previously reported cases [2-6]. Here we report acute hepatotoxicity after ZOL infusion, which did not recur after switching to another BP.

## Case Presentation

A 50-year-old female patient presented to the rheumatology clinic for osteoporosis management. Her past medical history was notable for invasive ductal carcinoma of her right breast, treated with surgery, chemotherapy, radiation, and then hormonal therapy with anastrozole and abemaciclib, to be taken for the next 10 years. About 4 months before starting anastrozole, she had a dual-energy x-ray absorptiometry scan indicating osteopenia, with T scores ranging from −1.9 to −2.4. Two weeks after starting her anastrozole prescription, she was first seen in our bone health clinic. Given her T scores in the osteopenic range while now on hormonal therapy, antiresorptive treatment was recommended. Despite counseling, the patient declined and proceeded first to try conservative measures. Her dual-energy x-ray absorptiometry scan 1 year later showed a 3.0% to 6.0% bone mineral density loss with T scores now from −2.2 to −2.7, moving her into the osteoporotic range. Now with osteoporosis while on an aromatase inhibitor, she agreed to antiresorptive therapy. After an extensive discussion of her options, including once every 6 months ZOL 4 mg intravenously, denosumab 60 mg subcutaneously every 6 months, or ZOL 5 mg intravenously once every 12 months, a compromise was made to start with ZOL 5 mg intravenously due to the cost of international travel and lodging, which was prohibitive for this patient [7, 8].

Within a few hours of receiving her first infusion of ZOL, the patient reported severe bone pain, abdominal pain, bloating, and nausea. She presented to the emergency department afebrile, normotensive, but tachycardic. She denied fevers, chills, and any history of drug or alcohol consumption.

## Diagnostic Assessment

**Figure 1.**
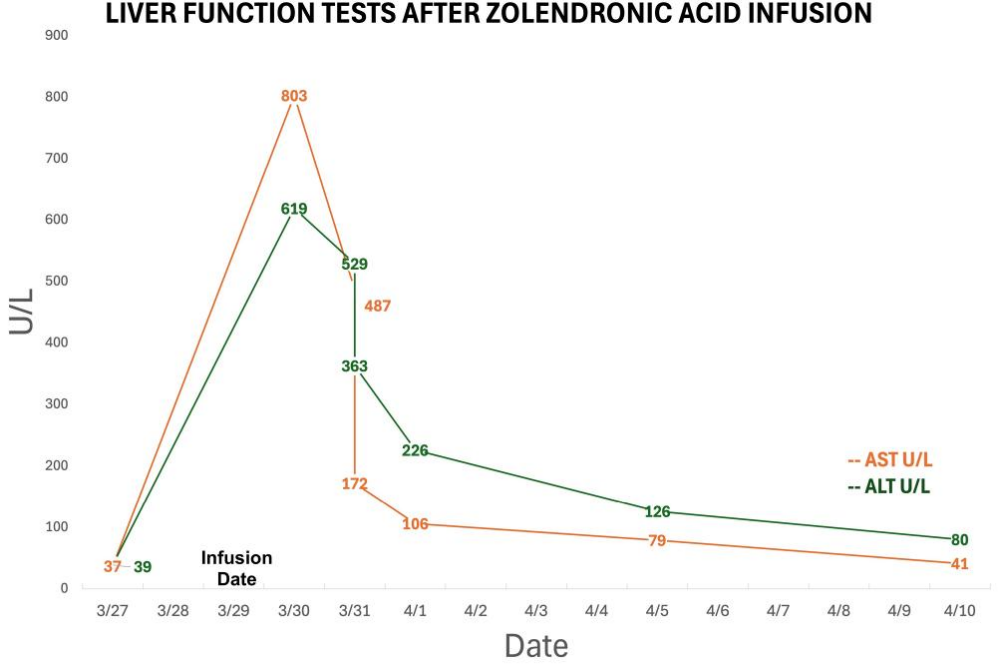
Liver function test elevation following zoledronic acid infusion.

Physical exam was notable for diffuse abdominal distention and tenderness without rebound or guarding. No jaundice or pruritus was appreciated. Laboratory tests were significant for an elevated transaminitis, alkaline phosphatase, and gamma-glutamyl transpeptidase; low phosphorus; normal bilirubin; normal amylase/lipase; normal creatine kinase; and normal calcium. Her serological testing for viral hepatitis was negative (Table 1). Before this event, her serum liver enzyme levels were always within normal range on routine screening tests (Fig. 1). A computed tomography scan of her abdomen/pelvis did not show any acute new findings. She had no prior history of liver disease and no evidence of metabolic dysfunction-associated steatotic liver disease on imaging. Her medications at that time included anastrozole, abemaciclib, vitamin D3, gabapentin, polyethylene glycol, and sennoside.

**Table 1.** Summary of zoledronic acid-induced liver injuries

Laboratory test	Units (conventional)	Units	Normal reference range, conventional (SI)
		(SI)	
Aspartate Aminotransferase	803 U/L	13.4 µkat/L	10-30 U/L (0-0.58 µkat/L)
Alanine aminotransferase	619 U/L	10.3 µkat/L	10-40 U/L (0.17-0.92 µkat/L)
Alkaline phosphatase	122 U/L	2.0 µkat/L	30-130 U/L (0.73-2.45 µkat/L)
Gamma-glutamyl transpeptidase	639 U/L	10.7 µkat/L	5-40 U/L (0.08-0.67 µkat/L)
Phosphorus	1.7 mg/dL	0.54 mmol/L	2.5-4.5 mg/dL (0.81-1.45 mmol/L)
Bilirubin (total)	0.8 mg/dL	13.6 µmol/L	0.1-1.2 mg/dL (1.7-20.5 µmol/L)
Amylase	70 U/L	1.17 µkat/L	40-140 U/L (0.38-1.42 µkat/L)
Lipase	50 U/L	0.84 µkat/L	0-160 U/L (0.17-2.35 µkat/L)
Creatine kinase	50 U/L	0.84 µkat/L	20-50 U/L (0.63-2.91 µkat/L)
Calcium	8.9 mg/dL	2.4 mmol/L	8.5-10.2 mg/dL (2.2-2.7 mmol/L)
Serological testing for viral hepatitis			
Anti-hepatitis A antibody	Negative	—	—
Hepatitis B surface antigen	Negative	—	—
Anti-hepatitis B surface antibody	Negative	—	—
Hepatitis B e antigen	Negative	—	—
Anti-hepatitis B e antibody	Negative	—	—
Anti-hepatitis B core antibody	Negative	—	—
Hepatitis B virus deoxyribonucleic acid	Negative	—	—
Anti-hepatitis C antibody	Negative	—	—
Hepatitis C virus ribonucleic acid	Negative	—	—

Abbreviations: SI, International System of Units.

## Treatment

The timing of the presentation and history suggested a hepatocellular drug-induced liver injury. The patient's pain and nausea improved with analgesics (ibuprofen 400 mg tablet every 6 hours as needed for mild pain and morphine sulfate immediate release 15 mg tablet every 4 hours as needed for moderate/severe pain) and antiemetics (first-line ondansetron 4 mg tablet every 4 hours as needed and second-line prochlorperazine IV push 5 mg every 6 hours as needed). Her liver function tests downtrended during her hospital admission (Fig. 1), and she was subsequently discharged when symptoms resolved. Anastrozole and abemaciclib were both held for 1 week while she was an inpatient and restarted on hospital discharge, and her liver function tests continued to downtrend as an outpatient.

## Outcome and Follow-up

Over the next month, her transaminitis gradually normalized. The decision was made to avoid ZOL in the future, and the patient was given alendronate a year later, which has been well tolerated. We discussed trialing denosumab, but since the patient lives overseas and could only visit our institution annually, she would have needed to find a local physician to treat her osteoporosis. Instead, she chose to start oral alendronate and follow up annually due to these logistical limitations. In addition, she will need antiresorptive therapy for more than 5 years as her anastrozole was planned for 10 years; thus the potential rebound bone loss and fracture risk after prolonged denosumab without close monitoring was a concern.

## Discussion

Fever, myalgia, and flu-like symptoms are some of the most common side effects after ZOL infusion. However, ZOL and other bisphosphonate-induced hepatotoxicity have rarely been reported [2-6, 9-11]. Up to this point, 5 previous cases have described liver injury following ZOL administration (Table 2): a 53-year-old female treated for Paget disease [2], a 73-year-old female treated for primary osteoporosis [3], a 50-year-old female with glucocorticoid-induced osteoporosis [4], and a 55-year-old with Sheehan syndrome [5]. In these cases, the patient's liver injury occurred less than 3 days after ZOL infusion, and liver function tests (LFTs) returned to baseline within 12 days. Interestingly, 1 case described ZOL-induced autoimmune hepatitis requiring short-term immunosuppression after LFTs did not downtrend after more than 72 hours [5]. In our case, liver injury occurred within a few hours of ZOL infusion, and LFTs reached normal limits after 11 days. Given the rapid resolution of her LFTs, autoimmune hepatitis testing and early glucocorticoid treatment was not done. However, further testing would have been warranted if the LFTs did not downtrend within 24 hours.

**Table 2.** Initial presenting laboratory values

	Year published	Age (years)/sex	Bisphosphonate	Underlying disease (PMH)	Time of first reported LFT elevation after starting therapy	Time to LFT normalization after discontinuation	Other complications
Case 1 [2]	2010	53/F	Zoledronic acid	Paget disease	24 hours	7 days	NA
Case 2 [3]	2013	73/F	Zoledronic acid	Primary osteoporosis	72 hours	12 days	NA
Case 3 [4]	2015	50/F	Zoledronic acid	Glucocorticoid-induced osteoporosis (Bechet's)	72 hours	9 days	NA
Case 4 [6]	2017	73/F	Zoledronic acid	Primary osteoporosis	7 days	91 days	Autoimmune hepatitis
Case 5 [5]	2022	55/F	Zoledronic acid	Sheehan syndrome	24 hours	6 days	NA
Our case	2024	50/F	Zoledronic acid	Aromatase inhibitor- induced osteoporosis (breast cancer)	12 hours	13 days	NA

Abbreviations: F, female; LFT, liver function test; NA, not applicable; PMH, past medical history.

Additionally, 3 prior cases have described liver injury following oral BP (alendronate and risedronate) administration [9-11]. In these cases, liver injury occurred 56 to 365 days after initial administration, then returned to baseline in 64 to 365 days. The difference in timing of liver injury due to oral BP is likely due to variable dosing compared to ZOL. There have been other cases of BP-induced hepatotoxicity reported with clodronate, risedronate, and ibandronate. In more than half of these cases, patients had a prior history of liver disease, and like our case, BP liver toxicity was mild to moderate [6].

Assessing preexisting risk factors, our patient had no known history of liver disease but was receiving concurrent immunotherapy with abemaciclib. Abemaciclib, a CDK-4 and CDK-6 inhibitor, can be a rare cause of liver injury. Taniguchi et al noted that patients taking abemaciclib while concurrently taking an aromatase inhibitor had an increased risk of liver injury [12]. Although our patient was taking both medications at the time, she had previously tolerated them for more than a year without hepatic injury until the administration of ZOL. The rapid, transient rise in LFTs following her first ZOL infusion is suggestive of BP-specific liver injury, which may have been augmented by taking abemaciclib and anastrozole.

The mechanism of BP-induced liver injury is not well understood. One mouse model study noted that ZOL induced apoptosis by increasing proinflammatory cytokines and oxidative stress, which can lead to subsequent liver damage [13]. Similarly, the transient increase in cytokines may explain the more common flu-like symptoms after ZOL infusion.

## Learning Points

- Hepatotoxicity is a rarely reported adverse effect of ZOL that resolved with discontinuation of the medication and did not recur after switching to another antiresorptive therapy.
- Patients with preexisting liver disease or taking concomitant hepatotoxic medications should be safely monitored for signs of liver damage.

## Contributors

All authors made individual contributions to authorship. H.L. was involved in diagnosing and managing the patient and reviewing the manuscript. C.B. and P.P. were engaged in writing and submitting the manuscript. All authors reviewed and approved the final draft.

## Data Availability

Data sharing is not applicable to this article as no datasets were generated or analyzed during the current study.
